# Agreement and utility of coded primary and secondary care data for long-term follow-up of clinical trial outcomes

**DOI:** 10.1186/s12874-025-02606-1

**Published:** 2025-06-07

**Authors:** Ariel Wang, Anna E Seeley, Matthew R Sydes, Nicholas Jones, Simon de Lusignan, FD Richard Hobbs, Richard J McManus, Marney Williams, James P Sheppard

**Affiliations:** 1 Nuffield Department of Primary Care Health Sciences, University of Oxford, Radcliffe Primary Care Building, Radcliffe Observatory Quarter, Oxford, OX2 6GG UK; 2 Data for R&D, Transformation Directorate, NHS England, London, UK; 3 MRC Clinical Trials Unit at UCL, Institute of Clinical Trials and Methodology, University College London, London, UK; 4 Brighton and Sussex Medical School, University of Brighton and University of Sussex, Brighton, UK; 5 Patient and public involvement representative, London, UK

**Keywords:** Electronic health records (EHRs), Routinely collected health data (RCHD), Healthcare systems data (HSD), Randomised controlled trial (RCT), Data utility, Data linkage, Data utility comparison studies (DUCkS), Primary care, Secondary care

## Abstract

**Background:**

Whilst interest in efficient trial design has grown with the use of electronic health records (EHRs) to collect trial outcomes, practical challenges remain. Commonly raised concerns often revolve around data availability, data quality and issues with data validation. This study aimed to assess the agreement between data collected on clinical trial participants from different sources to provide empirical evidence on the utility of EHRs for follow-up in randomised controlled trials (RCTs).

**Methods:**

This retrospective, participant-level data utility comparison study was undertaken using data collected as part of a UK primary care-based, randomised controlled trial (OPTiMISE). The primary outcome measure was the recording of all-cause hospitalisation or mortality within 3 years post-randomisation and was assessed across (1) Coded primary care data; (2) Coded-plus-free-text primary care data; and (3) Coded secondary care and mortality data. Agreement levels across data sources were assessed using Fleiss’ Kappa (K). Kappa statistics were interpreted using an established framework, categorising agreement strength as follows: <0 (poor), 0.00–0.20 (slight), 0.21–0.40 (fair), 0.41–0.60 (moderate), 0.61–0.80 (substantial), and 0.81–1.00 (almost perfect) agreement. The impact of using different data sources to determine trial outcomes was assessed by replicating the trial’s original analyses.

**Results:**

Almost perfect agreement was observed for mortality outcome across the three data sources (K = 0.94, 95%CI 0.91–0.98). Fair agreement (weak consistency) was observed for hospitalisation outcomes, including all-cause hospitalisation or mortality (K = 0.35, 95%CI 0.28–0.42), emergency hospitalisation (K = 0.39, 95%CI 0.33–0.46), and hospitalisation or mortality due to cardiovascular disease (K = 0.32, 95%CI 0.19–0.45). The overall trial results remained consistent across data sources for the primary outcome, albeit with varying precision.

**Conclusion:**

Significant discrepancies according to data sources were observed in recording of secondary care outcomes. Investigators should be cautious when choosing which data source(s) to use to measure outcomes in trials. Future work on linking participant-level data across healthcare settings should consider the variations in diagnostic coding practices. Standardised definitions for outcome measures when using healthcare systems data and using data from different data sources for cross-checking and verification should be encouraged.

**Supplementary Information:**

The online version contains supplementary material available at 10.1186/s12874-025-02606-1.

## Introduction

Well-designed and conducted randomised controlled trials (RCTs) are considered the gold standard for assessing the efficacy and safety of interventions [1, 2]. A key aspect of the good conduct of RCTs is the careful collection and validation of baseline and follow-up data. Ideally, follow-up data would be collected for a significant period of time, in order to capture as many clinical outcome events as possible. However, conducting long-term follow-up within trial is often restricted by time and cost [3]. Moreover, substantial logistical and methodological complexities such as participant retention, data completeness, and long-term follow-up feasibility add significant challenges in conducting such follow-ups [4, 5]. Over the past two decades, interest has mounted in utilising electronic health records (EHRs) to simplify components of RCTs [6, 7, 8, 9]. It was reported in a review that 113 studies published between 1945 and 2016, of which 71 (63%) were conducted after 2010, utilised administrative and registry data for long-term follow-up of participants in trials [6]. Despite the increasing numbers of studies that used EHRs for trial follow-up [7, 8, 9, 10], these numbers remain small when comparing to the backdrop of over 5,000 registered trials publishing their results every year [11].

Whilst greater use of EHR for clinical trial follow-up is advocated [12], practical challenges remain. Commonly raised concerns often revolve around data availability, data quality and issues with data validation [13]. Lensen et al. (2020) [9] reported that 30% (27/91) of studies in their review used EHRs for cross-checking trial data, while 57% (52/91) relied solely on EHRs as trial data without any cross-checking or comparison against other data sources. Previous studies have revealed significant disparities in recording of events in primary care, secondary care, disease registration and trial data [14, 15, 16, 17, 18]. Two studies, though, suggest that coded hospital admission and death registry data in the UK has the potential to be used as the sole method for collecting serious vascular outcomes without the need for verification by clinical adjudication [16, 17]. This data utility comparison study compared coded data from primary and secondary care, and where collected systematically for a trial [19, 20]. We aimed to assess how well the coded data from primary care and secondary care could be used to replace or supplement trial data, and examine the impact of different data sources on trial outcome assessment in a completed, UK primary care-based randomised controlled trial with outcome measures involving secondary care [19, 20].

## Methods

### Design

The present retrospective, participant-level data utility comparison study used data from a UK primary care-based, open-label, randomised controlled trial– OPtimising Treatment for MIld Systolic hypertension in the Elderly (OPTiMISE) [Trial registration ISRCTN 97503221, Registration date: 15/03/2017] [19]. The OPTiMISE trial was conducted in 69 primary care sites in England. It aimed to find out whether and to what extent blood pressure medications could be safely reduced in older patients (≥ 80 years old) with controlled blood pressure (systolic blood pressure < 150mmHg) who were on multiple antihypertensive medications. A total of 569 participants were randomised (1:1 ratio) to the trial, of whom 564 (99.1%) were followed up for at least 3 years after randomisation. Data used in this embedded methodological substudy was collected as part of the long-term follow-up of the OPTiMISE trial [20]. The overall trial was approved by a National Health Service (NHS) Research Ethics Committee (South Central—Oxford A; 16/SC/0628) and the Medicines and Healthcare products Regulatory Agency (MHRA; 21584/0371/001–0001). All participants gave written informed consent.

### Data sources

This substudy used three sources of data to assess study outcomes:

1. Coded primary care data– extracted directly from the Oxford Clinical Informatics Digital Hub (ORCHID) platform. ORCHID is a live database which includes nearly 2,000 practices and 20 million patient records (https://orchid.phc.ox.ac.uk) [21, 22]. It allows near real-time data collection from primary care EHRs including all coded data, prescriptions, test results and coded diagnoses.
2. Coded-plus-free-text primary care data– manually extracted by a member of the research team, who went to each study site and conducted systematic manual notes reviews for each participant in the trial.
3. Coded secondary care and mortality data– extracted from centralised datasets held by NHS England (NHSE), which included Hospital Episode Statistics (HES) Admitted Patient Care (APC), HES Accident and Emergency, and The Office for National Statistics (ONS) Death Registration Data (data sharing agreement DARS-NIC-459340-M8R2R-v0.11).

### Outcome measures

The primary analysis assessed the agreement in recording a composite outcome of all-cause hospitalisation or mortality within 3 years post-randomisation across the three data sources described above.

The secondary analysis assessed the agreement across the three data sources in recording all-cause mortality, emergency hospitalisation, and hospitalisation or death due to cardiovascular diseases (CVD), myocardial infarction (MI), stroke, and other serious adverse events (SAEs) resulting in hospitalisation including acute kidney injury (AKI), falls, fractures, hypotension, syncope and electrolyte abnormalities within 3 years post-randomisation. In this analysis, cause-specific hospitalisations were defined based on the primary diagnosis in the HES Admitted Patient Care dataset using prespecified ICD-10 codes documented in the electronic health records (see Appendix I in the supplementary document), whereas the sensitivity analysis incorporated both primary and secondary diagnoses. Cause-specific mortality was defined using both primary and secondary causes of death.

### Statistical methods

The central aim of this data utility comparison study was to assess the agreement of data collected from three data sources in identifying participants who experienced the designated outcome events among those for whom all sources were available. The performance of primary care data sources (Coded primary care data, and Coded-plus-free-text primary care data) were assessed using sensitivity and specificity against the reference standard (Coded secondary care and mortality data). The overall levels of agreement across data sources were estimated using the Cohen’s kappa (for two raters) and Fleiss’ kappa (for three raters) [23, 24, 25, 26]. Kappa statistics were interpreted using an established framework, categorising agreement strength as follows: <0 (poor), 0.00–0.20 (slight), 0.21–0.40 (fair), 0.41–0.60 (moderate), 0.61–0.80 (substantial), and 0.81–1.00 (almost perfect) agreement [27]. The agreement on the primary outcome (all-cause hospitalisation or mortality) was further evaluated within subgroups using heterogeneity testing. Analyses were stratified by treatment allocation (medication reduction vs. usual care), baseline frailty (electronic frailty index score ≤ 0·12 vs. > 0.12 [fit vs. frail]), baseline cognitive function (Montreal Cognitive Assessment [MoCA] score < 26 vs. ≥26), number of antihypertensive medications prescribed at baseline (2 vs. >2), and number of co-morbidities at baseline (≤ 4 morbidities vs. >4). To identify the cause-specific outcomes (secondary outcomes) within coded data from primary care (in ORCHID database), all hospitalisation events occurring during the 3-year follow-up period were reviewed by a general practitioner (AES) to determine whether a corresponding diagnosis code (separated data entry) could be identified for the same participant, within seven days of the hospitalisation date.

To determine whether using different data sources to define outcomes would yield similar treatment effect estimates as observed in the OPTiMISE long-term follow-up study, we replicated the original analyses using these three data sources. In this study, the coded secondary care and mortality data were the same as those in the original OPTiMISE trial dataset. A Cox proportional hazards model adjusting for baseline systolic blood pressure and treatment allocation as a fixed effect was used. All analyses carried out based on intention-to-treat (ITT) groups, at the 0.05 (2sided) significance level.

All data were analysed using Stata statistical software (Stata MP version 18.0) [28].

## Results

### Population characteristics

**Fig. 1:**
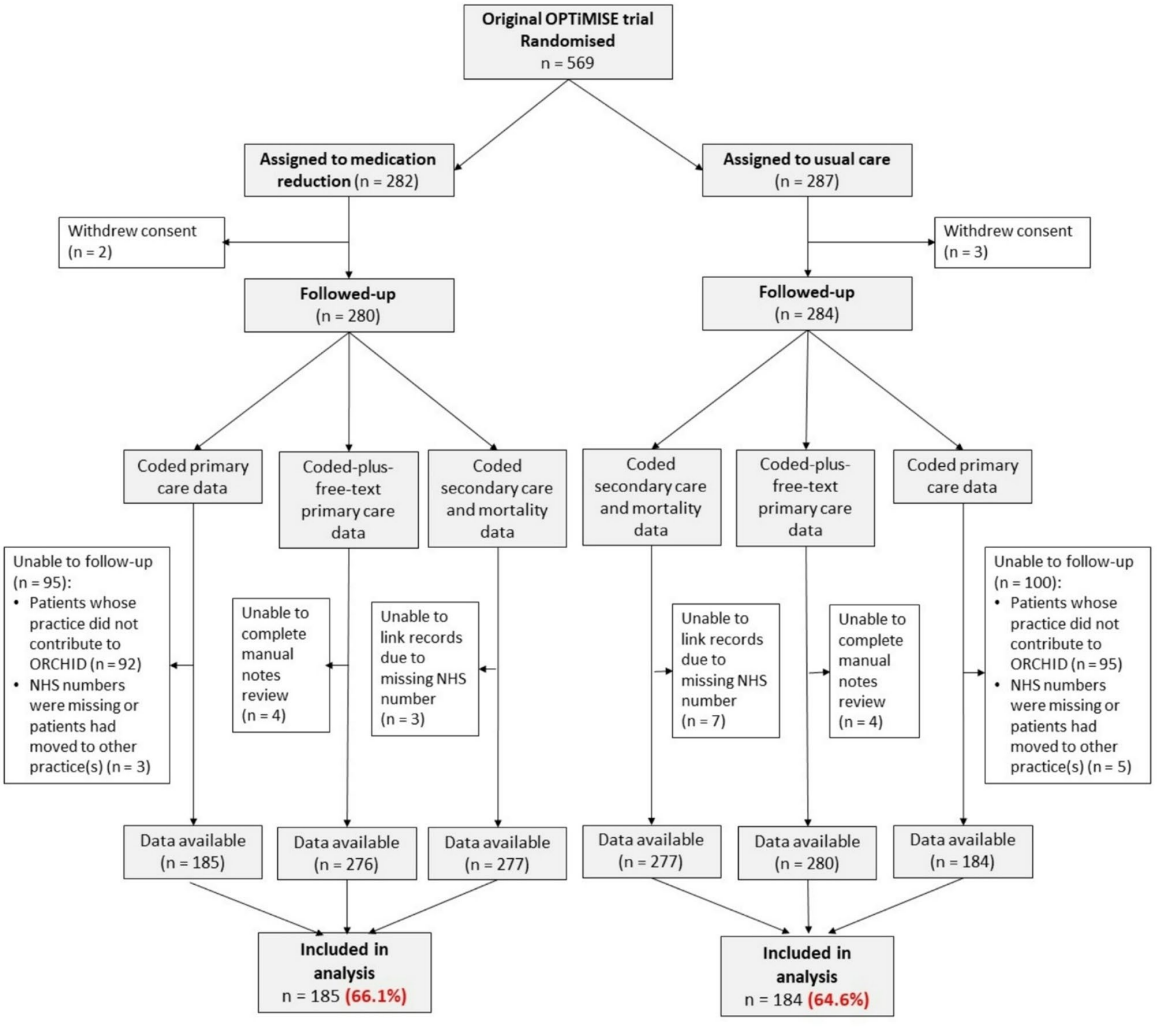
Data extraction flow diagram

A total of 569 participants were randomised in the original OPTiMISE trial. Of these, 187 patients were registered at practices that did not contribute to the ORCHID database so their coded primary care data were unavailable. Additionally, five participants withdrew consent for extended follow-up, and eight participants could not be linked to any of the data sources used for extended follow-up, because their NHS numbers were missing or they had moved to other practice(s) after randomisation. As a result, long-term follow-up data from all three sources were available for 369 (65%) trial participants (Fig. 1). A comparison of baseline characteristics showed no substantial differences between participants available for this analysis and the complete OPTiMISE trial cohort (Table 1).

**Table 1:** Baseline characteristics of participants, by treatment allocation, by source of data

	Data utility comparison substudy			Overall OPTiMISE trial		
	Medication reduction (*n* = 185)	Usual care (*n* = 184)	Total (*n* = 369)	Medication reduction (*n* = 280)	Usual care (*n* = 284)	Total (*n* = 564)
Age (mean, SD)	84.8 (3.6)	85.4 (3.7)	85.0 (3.7)	84.7 (3.3)	85.0 (3.6)	84.8 (3.4)
SBP (mean, SD)	128.9 (13.5)	131.1 (11.7)	130.0 (12.7)	129.4 (13.2)	130.5 (12.3)	130.0 (12.7)
DBP (mean, SD)	67.7 (9.2)	69.6 (8.5)	68.6 (8.9)	68.4 (9.1)	70.1 (8.4)	69.3 (8.8)
BMI (mean, SD)	26.9 (4.2)	27.8 (4.1)	27.3 (4.2)	27.2 (4.2)	28.0 (4.3)	27.6 (4.3)
eFI (mean, SD)	0.15 (0.07)	0.15 (0.07)	0.15 (0.07)	0.14 (0.07)	0.15 (0.07)	0.15 (0.07)
No. of antihypertensives (mean, SD)	2.5 (0.6)	2.5 (0.7)	2.5 (0.7)	2.5 (0.6)	2.5 (0.7)	2.5 (0.6)
No. of other medications (mean, SD)	3.3 (3.5)	3 (3.2)	3.1 (3.3)	2.8 (3.4)	2.7 (3.3)	2.8 (3.4)
Gender (n, %)						
Male	102 (55.1%)	95 (51.6%)	197 (53.4%)	150 (53.6%)	141 (49.6%)	291 (51.6%)
Female	83 (44.9%)	89 (48.4%)	172 (46.6%)	130 (46.4%)	143 (50.4%)	273 (48.4%)
Ethnicity (n, %)						
White	182 (98.4%)	175 (95.1%)	357 (96.7%)	273 (97.5%)	270 (95.1%)	357 (96.7%)
Black	2 (1.1%)	3 (1.5%)	5 (1.4%)	2 (1.1%)	3 (1.5%)	5 (1.4%)
Asian (South)	0	2 (1.1%)	2 (0.5%)	0	2 (1.1%)	2 (0.5%)
Mixed and other	0	2 (1.1%)	2 (0.5%)	0	2 (1.1%)	2 (0.5%)
Missing/Unknown	1 (0.45%)	2 (1.1%)	3 (0.8%)	1 (0.45%)	2 (1.1%)	3 (0.8%)
BMI groups (n, %)						
Underweight	0	2 (1.1%)	2 (0.5%)	1 (0.4%)	2 (0.7%)	3 (0.5%)
Normal	58 (31.4%)	41 (22.3%)	99 (26.8%)	80 (28.6%)	59 (20.8%)	139 (24.7%)
Overweight	85 (46%)	70 (38%)	155 (42%)	131 (46.8%)	121 (42.6%)	252 (44.7%)
Obese	32 (17.3%)	52 (28.3%)	841 (22.8%)	56 (20%)	79 (27.8%)	135 (23.9%)
Missing/Unknown	10 (5.4%)	19 (10.3%)	29 (7.9%)	12 (4.3%)	23 (8.1%)	35 (6.2%)
Smoking (n, %)						
Non-smoker	107 (57.8%)	105 (57.1%)	212 (57.5%)	163 (58.2%)	164 (57.8%)	327 (58%)
Ex-smoker	74 (40%)	71 (38.6%)	145 (39.3%)	110 (39.3%)	111 (39.1%)	221 (39.2%)
Current smoker	0	4 (2.2%)	4 (1.1%)	3 (1.1%)	5 (1.8%)	8 (1.4%)
Missing	4 (2.2%)	4 (2.2%)	8 (2.2%)	4 (1.4%)	4 (1.4%)	8 (1.4%)
eFI^1^ (n, %)						
Fit	71 (38.4%)	70 (38.0%)	141 (38.2%)	121 (43.2%)	109 (38.4%)	230 (40.8%)
Mild	96 (51.9%)	95 (51.6%)	191 (51.8%)	130 (46.4%)	140 (49.3%)	270 (47.9%)
Moderate	16 (8.7%)	18 (9.8%)	34 (9.2%)	27 (9.6%)	32 (11.3%)	59 (10.5%)
Severe	2 (1.1%)	1 (0.5%)	3 (0.8%)	2 (0.7%)	3 (1.1%)	5 (0.9%)
Comorbidities (n, %)						
Chronic kidney disease	56 (30.3%)	64 (34.8%)	120 (32.5%)	82 (29.3%)	102 (35.9%)	184 (32.6%)
Cancer	49 (26.5%)	45 (24.5)	94 (25.5%)	66 (23.6%)	68 (23.9)	134 (23.8%)
Cardiac disease	45 (24.3%)	42 (22.8%)	87 (23.6%)	60 (21.4%)	60 (21.1%)	120 (21.3%)
Myocardial infarction	13 (7%)	17 (9.2%)	30 (8.1%)	20 (7.1%)	19 (6.7%)	39 (6.9%)
Coronary heart disease	35 (18.9%)	33 (17.9%)	68 (18.4%)	48 (17.1%)	48 (16.9%)	96 (17%)
Angina	19 (10.3%)	20 (10.9%)	39 (10.6%)	28 (10%)	30 (10.6%)	58 (10.3%)
Heat failure	4 (2.2%)	3 (1.6%)	7 (1.9%)	5 (1.8%)	5 (1.8%)	10 (1.8%)
Diabetes (Type 2)	33 (17.8%)	24 (13%)	57 (15.5%)	48 (17.1%)	53 (18.7%)	101 (17.9%)
Atrial fibrillation	34 (18.4%)	27 (14.7%)	61 (16.5%)	45 (15.9%)	45 (16.1%)	90 (16%)
TIA	19 (10.3%)	11 (6%)	30 (8.1%)	27 (9.6%)	22 (7.8%)	49 (8.7%)
Stroke	17 (9.2%)	14 (7.6%)	31 (8.4%)	22 (7.9%)	22 (7.8%)	44 (7.8%)
PVD	5 (2.7%)	7 (3.8%)	12 (3.3%)	6 (2.1%)	9 (3.2%)	15 (2.7%)

Note: SBP = systolic blood pressure; DBP = diastolic blood pressure; eFI = electronic frailty index; TIA = transient ischemic attack; PVD = peripheral vascular disease

*1 The electronic frailty index includes 36 items and is estimated from electronic health records. The index ranges from 0 to 1 (“fit” 0 ≤ eFI ≤ 0.12; “mild” 0.12 < eFI ≤ 0.24; “moderate” 0.24 < eFI ≤ 0.36; “severe” 0.36 < eFI ≤ 1.0)*

### Agreement across data sources

**Fig. 2:**
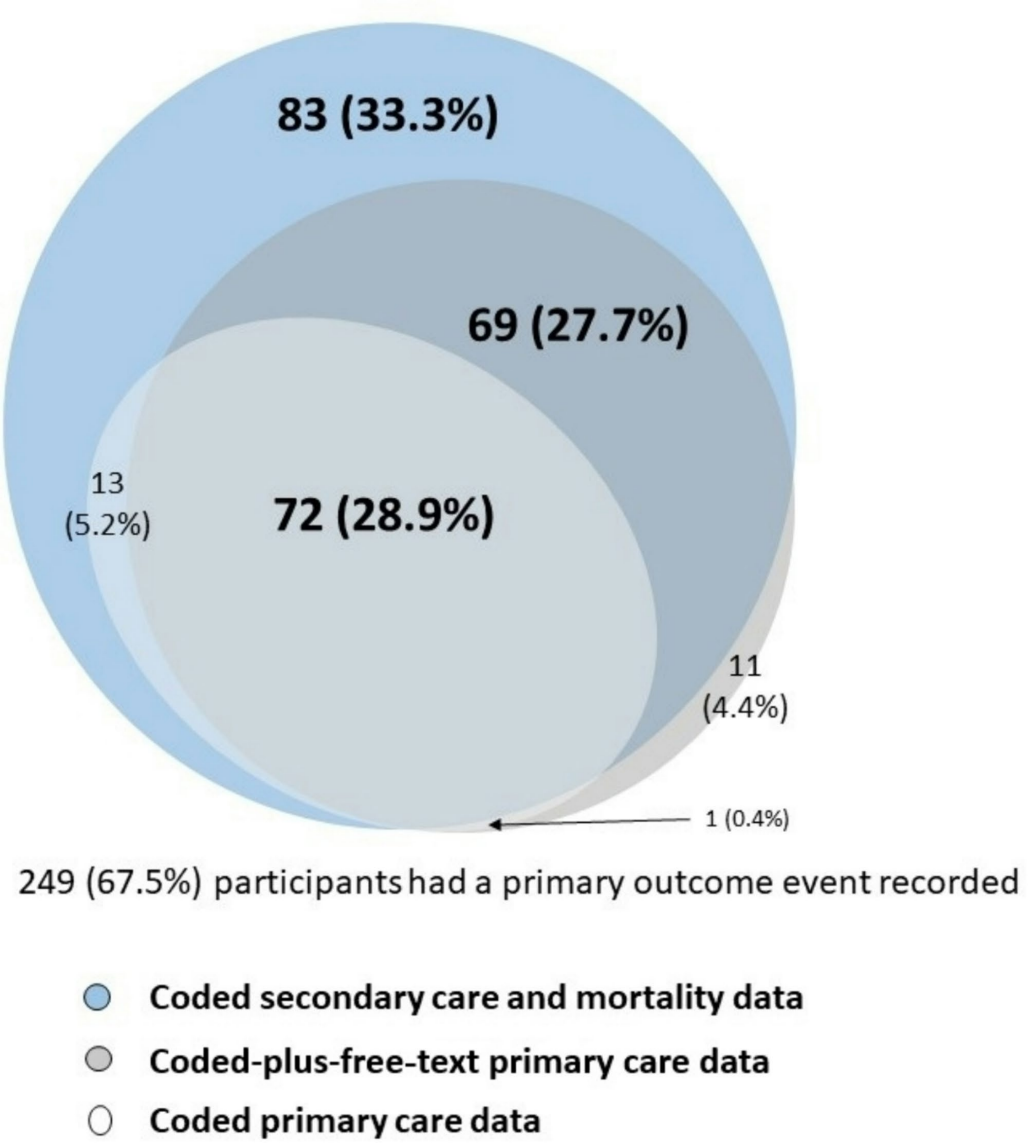
Incidence of all-cause hospitalisation and mortality during follow-up by source of data

The primary outcome measure (all-cause hospitalisation or mortality) was recorded in 249 (67.5%) participants in at least one data source. While 72 outcome events were identified consistently across all three data sources, 83 events were recorded only in coded secondary care and mortality data, and 11 events only in the coded-plus-freetext primary care data (Fig. 2). Compared to the reference standard (coded secondary care and mortality data), coded primary care data demonstrated low sensitivity meaning that only 35.9% (95% confidence interval [CI] 29.8–42.3%) of patients who experienced primary outcome events were recorded in coded primary care data. In contrast, specificity was high at 99.2% (95% CI 95.9–100%), meaning that nearly all patients who did not experience the primary outcome were accurately recorded as such. Coded-plus-free-text primary care data had higher sensitivity (59.5%; 95% CI 52.9–65.8%) but slightly lower specificity (90.9%; 95%CI 84.7–95.2%) (Table 2) compared to the reference standard (coded secondary care and mortality data).

**Table 2:** Primary and Secondary outcomes– only primary diagnosis was used to define the event specific hospitalisation (*N* = 369)

	Number of outcome events				Coded primary care data		Coded-plus-free-text primary care data		Kappa (95%CI)		
	Coded primary care data	Coded-plus-free-text primary care data	Secondary care and mortality data	In any of the data sources^1^	Sensitivity^2^ (95%CI)	Specificity (95%CI)	Sensitivity (95%CI)	Specificity (95%CI)	Coded primary care vs. secondary care	Coded-plus-free-text primary care vs. secondary care	Across all data sources^3^
All-cause hospitalisation or mortality	86 (23.3%)	153 (41.5%)	237 (64.2%)	249 (67.5%)	35.9% (29.8–42.3%)	99.2% (95.9–100%)	59.5% (52.9–65.8%)	90.9% (84.7–95.2%)	0.28 (0.22 to 0.34)	0.44 (0.36 to 0.52)	0.35 (0.28 to 0.42)
All-cause mortality	44 (11.9%)	51 (13.8%)	51 (13.8%)	51 (13.8%)	86.3% (73.7–94.3%)	100% (98.8–100%)	100% (93–100%)	100% (98.8–100%)	0.92 (0.85 to 0.98)	1	0.94 (0.91 to 0.98)
Emergency hospitalisation	39 (10.6%)	114 (30.9%)	124 (33.6%)	150 (40.7%)	27.4% (19.8–36.2%)	98% (95.3–99.3%)	71.8% (63.0–79.5%)	89.8% (85.3–93.3%)	0.31 (0.21 to 0.40)	0.63 (0.54 to 0.71)	0.39 (0.33 to 0.46)
Hospitalisation or death due to CVD	10 (2.7%)	20 (5.4%)	21 (5.7%)	35 (9.5%)	19% (5.4–41.9%)	98.3% (96.3–99.4%)	47.6% (25.7–70.2%)	97.1% (94.8–98.6%)	0.23 (0.02 to 0.44)	0.46 (0.26 to 0.65)	0.32 (0.19 to 0.45)
Hospitalisation or death due to MI	2 (< 1%)	7 (1.9%)	9 (2.4%)	11 (3.0%)	11.1% (0.3–48.2%)	99.7% (98.5–100%)	55.6% (21.2–86.3%)	99.4% (98–99.9%)	0.17 (-0.13 to 0.48)	0.62 (0.33 to 0.90)	0.44 (0.22 to 0.65)
Hospitalisation or death due to stroke	2 (< 1%)	10 (2.7%)	6 (1.6%)	14 (3.8%)	16.7% (0.4–64.1%)	99.7% (98.5–100%)	50.0% (11.8–88.2%)	98.1% (96.1–99.2%)	0.24 (-0.15 to 0.64)	0.36 (0.06 to 0.67)	0.21 (0.07 to 0.35)
Hospitalisation due to hypotension	0	0	2 (< 1%)	2 (< 1%)	-	-	-	-	0	0	-0.00 (-0.00 to 0.00)
Hospitalisation due to syncope	1 (< 1%)	2 (< 1%)	8 (2.2%)	11 (3.0%)	0% (0–36.9%)	99.7% (98.5–100%)	0% (0–36.9%)	99.4% (98.0–99.9%)	-0.00 (-0.01 to 0.00)	-0.01 (-0.02 to 0.00)	-0.01 (-0.01 to -0.00)
Hospitalisation due to falls	6 (1.6%)	25 (6.8%)	32 (8.7%)	45 (12.2%)	12.5% (3.5–29%)	99.4% (97.9–99.9%)	40.6% (23.7–59.4%)	96.4% (93.9–98.1%)	0.19 (0.02 to 0.36)	0.41 (0.24 to 0.58)	0.30 (0.17 to 0.43)
Hospitalisation due to fracture	6 (1.6%)	6 (1.6%)	13 (3.5%)	16 (4.3%)	46.2% (19.2–74.9%)	100% (99–100%)	23.1% (5.0–53.8%)	99.2% (97.6–99.8%)	0.62 (0.37 to 0.88)	0.30 (0.03 to 0.57)	0.43 (0.21 to 0.64)
Hospitalisation due to electrolyte abnormalities	2 (< 1%)	2 (< 1%)	7 (1.9%)	9 (2.4%)	0% (0–41%)	99.4% (98–99.9%)	28.6% (3.7–71.0%)	100% (99–100%)	-0.01 (-0.02 to 0.00)	0.44 (0.03 to 0.85)	0.18 (-0.01 to 0.36)
Hospitalisation due to AKI	0	2 (< 1%)	6 (1.6%)	7 (1.9%)	-	-	16.7% (0.4–64.1%)	99.7% (98.5–100%)	0	0.24 (-0.15 to 0.64)	0.12 (-0.08 to 0.32)
Diagnosis of dementia^4^	10 (2.7%)	9 (2.4%)	-	10 (2.7%)	-	-	-	-	Coded primary care vs. Coded-plus-free-text primary care: 0.84 (0.66 to 1.00)		

Note: (1) Recording of outcome events in any of the three data sources; (2) Record of events from Secondary care and mortality data as reference standard; (3) Fleiss’ Kappa was used to examine the agreement across three data sources; (4) Diagnosis of dementia only available from primary care datasets (Coded primary care vs. Coded-plus-free-text primary care)

Overall agreement across all three data sources on the primary outcome (all-cause hospitalisation or mortality) was fair (Fleiss’ kappa [K] = 0.35, 95% CI 0.28–0.42) suggesting that the level of consistency was above chance but still relatively weak. In addition, lower agreement observed in coded primary care data (Cohen’s kappa = 0.28, 95% CI 0.22–0.34) compared to coded-plus-freetext primary care data (Cohen’s kappa = 0.44, 95% CI 0.36–0.52) suggesting that incorporating free-text data improved the consistency of recorded outcomes. Subgroup analyses suggested a potential lower level of agreement among patients with higher baseline frailty (K = 0.30, 95% CI 0.22–0.39) and multiple comorbidities (K = 0.29, 95% CI 0.19–0.39) (Supplementary Table S1).

All-cause mortality was recorded in 44/369 (11.9%) participants using coded primary care data, and in 51/369 (13.8%) participants using coded-plus-freetext primary care data or coded secondary care and mortality data (Table 2). There was near-perfect agreement in mortality recording across the three data sources (K = 0.94, 95%CI 0.91–0.98).

Meanwhile, fair to moderate levels of agreement were observed for hospitalisation outcomes, including hospitalisation or mortality due to CVD (K = 0.32, 95%CI 0.19–0.45), stroke (K = 0.21, 95%CI 0.07–0.35), and MI (K = 0.44, 95%CI 0.22–0.65), and hospitalisation due to falls (K = 0.30, 95%CI 0.17–0.43) and fractures (K = 0.43, 95%CI 0.21–0.64). Poor agreement was observed in relation to hospitalisations due to hypotension, syncope, electrolyte abnormalities and AKI.

Sensitivity analyses indicated that including both primary and secondary diagnoses to define cause-specific hospitalisations resulted in lower levels of agreement particularly for hospitalisations due to electrolyte abnormalities and AKI, with the exception of hospitalisation or mortality due to stroke (Supplementary Table S2). Supplementary Figures S1 and S2 demonstrate that using both primary and secondary causes of hospitalisation led to a striking increase in the total number of CVD events recorded.

### Trial results comparison using routinely-collected primary care data

For the primary outcome and most secondary outcomes of interest, despite substantial differences in event rate, the point estimates of treatment effect were similar across the data sources (Table 3). For secondary outcome measures such as emergency hospitalisation, MI, falls and fracture, the direction of effect varied by data source, although this was likely due to the small numbers of events in most cases. Overall, 120/185 (64.9%) patients in the trial’s Medication Reduction group and 117/184 (63.6%) in the trial’s Usual Care group experienced all-cause hospitalisation or mortality (adjusted hazard ratio [aHR] = 1.04; 95% CI 0.81–1.35). Fewer events were recorded in coded-plus-freetext primary care data (medication reduction 42.2% vs. usual care 40.8%; aHR = 1.02; 95% CI 0.74–1.40; Table 3), and even fewer in coded primary care data (24.3% vs. 22.3% respectively; aHR = 1.08; 95% CI 0.70–1.65; Table 3). With fewer events, the confidence intervals around these similar point estimates were wider, with implications for statistical testing. There was no evidence of statistical significant differences between groups in the point estimates for any prespecified time-to-event secondary outcomes (Table 3). In the sensitivity analysis, using all available NHSE diagnostic codes increased the event rate, but did not substantially change point estimates, nor result in different findings across the data sources.

**Table 3:** Time to event analyses of clinical outcomes at follow-up (Data available from both datasets, *N* = 369)

	Medication reduction group	Usual care Group	Adjusted hazard ratio^1^ (95% CI)	*P*-value
Primary outcome (all-cause hospitalisation or mortality)				
Coded primary care data	45/185 (24.3%)	41/184 (22.3%)	1.08 (0.70 to 1.65)	0.733
Coded-plus-free-text primary care data	78/185 (42.2%)	75/184 (40.8%)	1.02 (0.74 to 1.40)	0.903
Coded secondary care and mortality data^2^	120/185 (64.9%)	117/184 (63.6%)	1.04 (0.81 to 1.35)	0.755
Secondary outcomes				
All-cause mortality				
Coded primary care data	22/185 (11.9%)	22/184 (12%)	0.96 (0.53 to 1.75)	0.899
Coded-plus-free-text primary care data	24/185 (13%)	27/184 (14.7%)	0.86 (0.49 to 1.49)	0.584
Coded secondary care and mortality data	24/185 (13%)	27/184 (14.7%)	0.85 (0.49 to 1.48)	0.569
Emergency hospitalisation				
Coded primary care data	23/185 (12.4%)	16/184 (8.7%)	1.34 (0.70 to 2.55)	0.375
Coded-plus-free-text primary care data	58/185 (31.4%)	56/184 (30.4%)	0.99 (0.69 to 1.44)	0.966
Coded secondary care and mortality data	69/185 (37.3%)	55/184 (29.9%)	1.27 (0.89 to 1.89)	0.190
Hospitalisation or death due to cardiovascular diseases				
Coded primary care data	5/185 (2.7%)	5/184 (2.7%)	0.89 (0.25 to 3.11)	0.850
Coded-plus-free-text primary care data	9/185 (4.9%)	11/184 (6%)	0.79 (0.33 to 1.92)	0.605
Coded secondary care and mortality data	8/185 (4.3%)	13/184 (7.1%)	0.56 (0.23 to 1.36)	0.199
Hospitalisation or death due to myocardial infarction				
Coded primary care data	1/185 (< 1%)	1/184 (< 1%)	1.06 (0.07 to 16.93)	0.969
Coded-plus-free-text primary care data	4/185 (2.2%)	3/184 (1.6%)	1.27 (0.28 to 5.74)	0.755
Coded secondary care and mortality data	3/185 (1.6%)	6/184 (3.3%)	0.41 (0.10 to 1.69)	0.219
Hospitalisation or death due to stroke				
Coded primary care data	1/185 (< 1%)	1/184 (< 1%)	0.66 (0.04 to 11.61)	0.776
Coded-plus-free-text primary care data	3/185 (1.6%)	7/184 (3.8%)	0.41 (0.11 to 1.62)	0.205
Coded secondary care and mortality data	2/185 (1.1%)	4/184 (2.2%)	0.46 (0.08 to 2.53)	0.370
Hospitalisation due to hypotension				
Coded primary care data	0	0	-	-
Coded-plus-free-text primary care data	0	0	-	-
Coded secondary care and mortality data	1/185 (< 1%)	1/184 (< 1%)	0.99 (0.06 to 15.97)	0.992
Hospitalisation due to syncope				
Coded primary care data	1/185 (< 1%)	0/184 (0%)	-	-
Coded-plus-free-text primary care data	2/185 (1.1%)	0/184 (0%)	-	-
Coded secondary care and mortality data	4/185 (2.2%)	4/184 (2.2%)	1.01 (0.25 to 4.04)	0.994
Hospitalisation due to falls				
Coded primary care data	3/185 (1.6%)	3/184 (1.6%)	1.06 (0.21 to 5.25)	0.945
Coded-plus-free-text primary care data	12/185 (6.5%)	13/184 (7.1%)	0.90 (0.41 to 1.99)	0.802
Coded secondary care and mortality data	18/185 (9.7%)	14/184 (7.6%)	1.27 (0.63 to 2.56)	0.508
Hospitalisation due to fracture				
Coded primary care data	5/185 (2.7%)	1/184 (< 1%)	5.03 (0.59 to 43.29)	0.141
Coded-plus-free-text primary care data	2/185 (1.1%)	4/184 (2.2%)	0.38 (0.07 to 2.18)	0.280
Coded secondary care and mortality data	10/185 (5.4%)	3/184 (1.6%)	3.41 (0.94 to 12.43)	0.063
Hospitalisation due to electrolyte abnormalities				
Coded primary care data	2/185 (1.1%)	0/184 (< 1%)	-	-
Coded-plus-free-text primary care data	1/185 (< 1%)	1/184 (< 1%)	1.06 (0.07 to 16.99)	0.966
Coded secondary care and mortality data	4/185 (2.2%)	3/184 (1.6%)	1.42 (0.32 to 6.36)	0.644
Hospitalisation due to acute kidney injury				
Coded primary care data	0	0	-	-
Coded-plus-free-text primary care data	1/185 (< 1%)	1/184 (< 1%)	0.84 (0.05 to 14.15)	0.904
Coded secondary care and mortality data	4/185 (2.2%)	2/184 (1.1%)	2.07 (0.38 to 11.32)	0.402
Diagnosis of dementia^3^				
Coded primary care data	6/185 (3.2%)	4/184 (2.2%)	1.29 (0.36 to 4.66)	0.698
Coded-plus-free-text primary care data	6/185 (3.2%)	3/184 (1.6%)	1.71 (0.42 to 6.98)	0.454

Note:

^1^Cox proportional hazards model adjusting baseline systolic blood pressure and intervention group as fixed effects. Hazard ration (HR) < 1 indicates favour to medication reduction group

^2^In this study, the Coded secondary care and mortality data represents the original OPTiMISE trial dataset

^3^Diagnosis of dementia only available from Primary care datasets (Coded primary care data vs. Coded-plus-free-text primary care data)

## Discussion

### Summary of main findings

This embedded methodological study assessed the agreement of data collected from three sources: coded primary care data, coded-plus-freetext primary care data, and coded secondary care and mortality data. Only fair-to-moderate agreement was found for hospitalisation outcomes, including the primary outcome measure of all-cause hospitalisation or mortality. High level of agreement was observed for mortality outcome. Additionally, poor agreement was observed for some pre-specified secondary outcome measure including cause-specific hospitalisations due to hypotension, syncope, electrolyte abnormalities and AKI. Sensitivity analyses suggested that these substantial discrepancies were more likely due to different coding practices and the intended purposes within primary and secondary care, especially for transient events or those occurring alongside other conditions deemed more critical for ongoing care [29, 30]. Therefore, while including both coded primary and secondary causes of hospitalisation increased the number of events consistently reported across multiple data sources this approach also reduced the overall agreement across data sources, lowered primary care coding sensitivity, and inflated the event rate by capturing potentially unrelated events.[30].

Despite coded-plus-freetext primary care data missing a substantial number of events, comparing the primary outcome between groups produced similar results regardless of the data source used, although using data from a single primary care source resulted in significantly less precision. This suggests careful consideration must be given when choosing data source(s) to capture the desired outcomes. For instance, relying on primary care records for outcomes predominantly recorded in secondary care, or vice-versa, may lead to less precise treatment effect estimates in RCTs.

### Strengths and limitations

There are notable limitations in this study. The small sample size, including the exclusion of 187 patients whose practices were not registered with the ORCHID database, reduced the statistical power and made subgroup assessments challenging. Additionally, the limited number of cause-specific secondary outcome measures reduced the precision of treatment effect estimates and the reliability of comparisons across data sources. In this study, we found that using different data sources yielded similar treatment effect estimates, although these results were not statistically significant. However, the choice of data source may influence study findings, particularly in small studies where statistically significant results are based on a limited number of outcome events. Reliance on coded EHR data meant that we were unable to determine whether discrepancies in outcome recording were due to missing documentation in a given dataset or the exclusion of relevant codes needed to capture the outcome. The general practitioner determined a cause-specific hospitalisation event in coded primary care data only if both hospitalisation and event diagnosis codes were recorded, but we may have missed events with only one type of code. Establishing a definitive reference standard was also challenging. We used coded secondary care and mortality data as the reference standard for acute severe conditions resulting in hospitalisation. However, each HES APC episode has one primary diagnosis and up to nineteen secondary diagnoses. While the primary diagnosis reflects the main reason for admission, secondary diagnoses may include comorbidities or chronic conditions related to the patient’s care [31]. Therefore, the incidence of some events may have been overestimated in hospital records. To mitigate this, we used only the primary cause of hospitalisation in main analyses, though we may have missed events recorded under multiple codes.

### Comparison with previous literature

Previous studies in the UK have identified considerable disparities in outcome events recording across different data sources. Wood et al. (2021) [32] reported that among 53.3 million individuals included in the NHS Digital Trusted Research Environment (TRE) for England, 30% of first-ever incident stroke or transient ischaemic attack were recorded exclusively in primary care, 29% exclusively in secondary care or mortality register, and only 41% were recorded across all data sources. Herrett et al. (2013) [14] compared the diagnostic validity of recording acute MI events in primary care, secondary care and disease registry. They found that relying on a single data source underestimated the crude incidence of acute MI by 25–50% compared with using multiple sources, including data from a disease specific registry. Other studies also reported that only 30–50% of matched records were found across different health systems [15, 16, 17, 18]. Nonetheless, mortality records were accurately recorded across primary care and mortality register, despite some reporting delays [33]. Harper et al. (2023) [16] observed moderate agreement between data sources, showing that the outcome events ascertained *via* UK routine data sources provided relative and absolute treatment effects consistent with trial adjudicated follow-up. In line with these findings, our study comparing coded data from primary care and secondary care also found substantial disparities in hospitalisation records. The primary outcome and most secondary outcomes identified through coded (plus-free-text) primary care data produced treatment effect estimates similar to those in the original OPTiMISE trial, although using data from a single primary care source without including secondary care data could result in less precise treatment effect estimates. In our sensitivity analysis, incorporating both primary and secondary diagnoses improved agreement in hospitalisation records for stroke but not for CVD, which in our study encompassed stroke, MI, and heart failure (HF). This finding aligns with a systematic review showing that while EHRs are generally reliable for research purpose, validity estimates for HF outcomes were low [34].

### Implications for trial delivery

This study provides evidence describing how well the data from primary care, and secondary care and mortality registers agree with one another. Our findings highlight the need for clear outcome definitions and validated, pre-specified code lists when assessing outcome events using EHRs for long-term follow-up of RCTs [35, 36]. Future research utilising primary care EHRs should be mindful of the differences in diagnostic coding practices between primary and secondary care systems. In addition, investigators should carefully consider which data source(s) to use for measuring trial outcomes. Researchers intending to use primary care EHRs solely to collect outcomes that usually result in hospitalisation should be aware of the potential risk of underestimation. National initiatives—such as plans for a single, integrated patient record—may improve data interoperability and help reduce such discrepancies in the future [37].

The findings of this study are important, given the increasing number of studies that have relied on EHRs to assess outcome events at follow-up since the Covid-19 pandemic, most notably the RECOVERY trial, the PRINCIPLE and PANORAMIC trial of treatments for COVID-19 [38, 39, 40]. If such data are not appropriate, this could impact our understanding of new treatments and their generalisability in the community. Our findings suggest that although significant disparities in recording of outcome events were observed, these were equally prevalent across both intervention groups, so the overall findings remained similar, albeit with less precise point estimates. However, due to the low number of events observed in our study, particularly for cause-specific secondary outcomes, the reliability of using single data source for collecting significant acute clinical outcomes remain uncertain. Researchers in this setting may wish to use linked primary care and secondary care data for cross-checking and verification.

## Conclusions

This study provides empirical evidence on the level of agreement across three data sources: coded primary care data, coded-plus-freetext primary care data, and coded secondary care and mortality data. Our findings suggest substantial discrepancies in hospitalisation records across primary and secondary care, which could affect the precision with which outcome events can be determined if used for follow-up in RCTs. Future work on linking participant-level data across healthcare settings should consider the variations in diagnostic coding practices, and using data from multiple data sources for cross-checking and verification.

## Electronic supplementary material

Below is the link to the electronic supplementary material.

Supplementary Material 1

## Data Availability

No datasets were generated or analysed during the current study.

## Declarations

### Data Sharing

Data sharing statement: Requests for sharing of de-identified individual participant data of the OPTiMISE trial and a data dictionary defining each field in the set will be considered by the corresponding author. The OPTiMISE study protocol is available on the trial website https://www.phctrials.ox.ac.uk/studies/optimise.
