# Supplementary material for: Agreement and utility of coded primary and secondary care data for long-term follow-up of clinical trial outcomes
Source: BMC Med Res Methodol. 2025 Jun 7;25:156. doi: 10.1186/s12874-025-02606-1 (PMC12144831; doi:10.1186/s12874-025-02606-1)
Supplement: Supplementary file 1 — Supplementary Material 1 [file 12874_2025_2606_MOESM1_ESM.docx]

*Supplementary Table S1. Subgroups analyses of the primary outcome – All-cause hospitalisation or mortality (N=369)*

|  | Number of outcome events | | | | Coded primary care data | | Coded-plus-free-text primary care data | | Kappa (95%CI) | | |  |
| --- | --- | --- | --- | --- | --- | --- | --- | --- | --- | --- | --- | --- |
|  | Coded primary care data | Coded-plus-free-text primary care data | Secondary care and mortality data | In any of the data sources^1^ | Sensitivity^2^ (95%CI) | Specificity (95%CI) | Sensitivity (95%CI) | Specificity (95%CI) | Coded primary care vs secondary care | Coded-plus-free-text primary care vs secondary care | Across all data sources^3^ |  |
| Allocation groups | | | |  |  |  |  |  |  |  |  |  |
| Medication reduction | 45 (52.3%) | 78 (51%) | 120 (50.6%) | 127 (51%) | 36.7% (28.1% to 45.9%) | 98.5% (91.7% to 100%) | 59.2% (49.8% to 68.0%) | 89.2% (79.1% to 95.6%) | 0.28 (0.19 to 0.37) | 0.42 (0.31 to 0.54) | 0.34 (0.24 to 0.44) |  |
| Usual care | 41 (47.7%) | 75 (49%) | 117 (49.4%) | 122 (49%) | 35% (26.5% to 44.4%) | 100% (94.6% to 100%) | 59.8% (50.4% to 68.8%) | 92.5% (83.4% to 97.5%) | 0.28 (0.20 to 0.37) | 0.46 (0.35 to 0.57) | 0.35 (0.28 to 0.48) |  |
| Baseline No. of antihypertensives | | | |  |  |  |  |  |  |  |  |  |
| 2 | 39 (45.3%) | 80 (52.3%) | 126 (53.2%) | 132 (53%) | 30.2% (22.3% to 39%) | 98.8% (93.3% to 100%) | 58.7% (49.6% to 67.4%) | 92.6% (84.6% to 97.2%) | 0.24 (0.16 to 0.32) | 0.47 (0.36 to 0.57) | 0.32 (0.23 to 0.41) |  |
| >2 | 47 (54.7%) | 73 (47.7%) | 111 (46.8%) | 117 (47%) | 42.3% (33% to 52.1%) | 100% (93% to 100%) | 60.4% (50.6% to 69.5%) | 88.2% (76.1% to 95.6%) | 0.32 (0.22 to 0.41) | 0.40 (0.28 to 0.53) | 0.37 (0.27 to 0.48) |  |
| Baseline frailty |  |  |  |  |  |  |  |  |  |  |  |  |
| Fit (eFI≤0.12) | 32 (37.2%) | 50 (32.7%) | 81 (34.2%) | 85 (34.1%) | 38.3% (27.7% to 49.7%) | 98.3% (91.1% to 100%) | 56.8% (45.3% to 67.8%) | 93.3% (83.8% to 98.2%) | 0.33 (0.22 to 0.45) | 0.47 (0.34 to 0.60) | 0.41 (0.30 to 0.53) |  |
| Frail (eFI>0.12) | 54 (62.8%) | 103 (67.3%) | 156 (65.8%) | 164 (65.9%) | 34.6% (27.2% to 42.6%) | 100% (95% to 100%) | 60.9% (52.8% to 68.6%) | 88.9% (79.3% to 95.1%) | 0.25 (0.18 to 0.32) | 0.42 (0.31 to 0.52) | 0.30 (0.22 to 0.39) |  |
| Baseline No. of co-morbidities | | | |  |  |  |  |  |  |  |  |  |
| 0-4 | 39 (45.3%) | 70 (45.8%) | 115 (48.5%) | 121 (48.6%) | 33% (24.6% to 42.4%) | 98.8% (93.6% to 100%) | 55.7% (46.1% to 64.9%) | 92.9% (85.3% to 97.4%) | 0.29 (0.20 to 0.38) | 0.45 (0.35 to 0.56) | 0.38 (0.28 to 0.48) |  |
| >4 | 47 (54.7%) | 83 (54.2%) | 122 (51.5%) | 128 (51.4%) | 38.5% (29.9% to 47.8%) | 100% (92.5% to 100%) | 63.1% (53.9% to 71.7%) | 87.2% (74.3% to 95.2%) | 0.26 (0.17 to 0.34) | 0.40 (0.28 to 0.52) | 0.29 (0.19 to 0.39) |  |
| Baseline cognitive function | | | |  |  |  |  |  |  |  |  |  |
| Normal (MoCA≥26) | 25 (29.1%) | 50 (32.7%) | 88 (37.1%) | 94 (37.8%) | 28.4% (19.3% to 39.0%) | 100% (93.3% to 100%) | 50% (39.1% to 60.9%) | 88.7% (77% to 95.7%) | 0.23 (0.14 to 0.32) | 0.34 (0.21 to 0.47) | 0.25 (0.14 to 0.37) |  |
| Impairment (MoCA<26) | 59 (68.6%) | 100 (65.4%) | 145 (61.2%) | 151 (60.6%) | 40% (32% to 48.5%) | 98.7% (93% to 100%) | 64.8% (56.5% to 72.6%) | 92.2% (83.8% to 97.1%) | 0.31 (0.22 to 0.39) | 0.50 (0.40 to 0.61) | 0.39 (0.31 to 0.48) |  |

*Note: 1. Recording of outcome events in any of the three data sources; 2. Record of events from Secondary care and mortality data as reference standard; 3. Fleiss' Kappa was used to examine the agreement across three data sources*

*Table S2. Sensitivity analysis – secondary outcomes, where both primary and secondary diagnoses were used to define the cause-specific hospitalisation (N=369)*

|  | Number of outcome events | | | | Coded primary care data | | Coded-plus-free-text primary care data | | Kappa (95%CI) | | |
| --- | --- | --- | --- | --- | --- | --- | --- | --- | --- | --- | --- |
|  | Coded primary care data | Coded-plus-free-text primary care data | Secondary care and mortality data | In any of the data sources^1^ | Sensitivity^2^ (95%CI) | Specificity (95%CI) | Sensitivity (95%CI) | Specificity  (95%CI) | Coded primary care vs secondary care | Coded-plus-free-text primary care vs secondary care | Across all data sources^3^ |
| Hospitalisation or death due to CVD | 10 (2.7%) | 20 (5.4%) | 51 (13.8%) | 57 (15.4%) | 15.7% (7% to 28.6%) | 99.4% (97.7% to 99.9%) | 31.4% (19.1% to 45.9%) | 98.7% (96.8% to 99.7%) | 0.23 (0.09 to 0.37) | 0.40 (0.26 to 0.55) | 0.29 (0.18 to 0.41) |
| Hospitalisation or death due to MI | 2 (<1%) | 7 (1.9%) | 17 (4.6%) | 17 (4.6%) | 11.8% (1.5% to 36.4%) | 100% (99% to 100%) | 41.2% (18.4% to 67.1%) | 100% (99% to 100%) | 0.20 (-0.04 to 0.44) | 0.57 (0.33 to 0.81) | 0.41 (0.19 to 0.62) |
| Hospitalisation or death due to stroke | 2 (<1%) | 10 (2.7%) | 11 (3%) | 16 (4.3%) | 9.1% (0.2% to 41.3%) | 99.7% (98.5% to 100%) | 54.5% (23.4% to 83.3%) | 98.9% (97.2% to 99.7%) | 0.15 (-0.11 to 0.41) | 0.56 (0.30 to 0.82) | 0.29 (0.17 to 0.41) |
| Hospitalisation due to hypotension | 0 | 0 | 20 (5.4%) | 20 (5.4%) | - | - | - | - | 0 | 0 | -0.02 (-0.03 to -0.01) |
| Hospitalisation due to syncope | 1 (<1%) | 2 (<1%) | 10 (2.7%) | 13 (3.5%) | 0% (0% to 30.8%) | 99.7% (98.5% to 100%) | 0% (0% to 30.8%) | 99.4% (98.0% to 99.9%) | -0.01 (-0.01 to 0.00) | -0.01 (-0.02 to 0.00) | -0.01 (-0.02 to -0.01) |
| Hospitalisation due to falls | 6 (1.6%) | 25 (6.8%) | 33 (8.9%) | 46 (12.5%) | 12.1% (3.4% to 28.2%) | 99.4% (97.9% to 99.9%) | 39.4% (22.9% to 57.9%) | 96.4% (93.8% to 98.1%) | 0.18 (0.02 to 0.35) | 0.40 (0.23 to 0.57) | 0.29 (0.16 to 0.42) |
| Hospitalisation due to fracture | 6 (1.6%) | 6 (1.6%) | 14 (3.5%) | 17 (4.6%) | 42.9% (17.7% to 71.1%) | 100% (99% to 100%) | 21.4% (4.7% to 50.8%) | 99.2% (97.6% to 99.8%) | 0.59 (0.33 to 0.85) | 0.28 (0.02 to 0.55) | 0.41 (0.20 to 0.62) |
| Hospitalisation due to electrolyte abnormalities | 2 (<1%) | 2 (<1%) | 37 (10%) | 38 (10.3%) | 2.7% (0.1% to 14.2%) | 99.7% (98.3% to 100%) | 5.4% (0.7% to 18.2%) | 100% (98.9% to 100%) | 0.04 (-0.05 to 0.13) | 0.09 (-0.03 to 0.21) | 0.04 (-0.04 to 0.11) |
| Hospitalisation due to AKI | 0 | 2 (<1%) | 30 (8.1%) | 31 (8.4%) | - | - | 3.3% (0.1% to 17.2%) | 99.7% (98.4% to 100%) | 0 | 0.05 (-0.06 to 0.16) | 0.00 (-0.06 to 0.06) |

*Note: 1. Recording of outcome events in any of the three data sources; 2. Record of events from Secondary care and mortality data as reference standard; 3. Fleiss' Kappa was used to examine the agreement across three data sources*

*Figure S1. Data sources recording participants’ CVD event – secondary analysis (A) vs sensitivity analysis (B)*

*
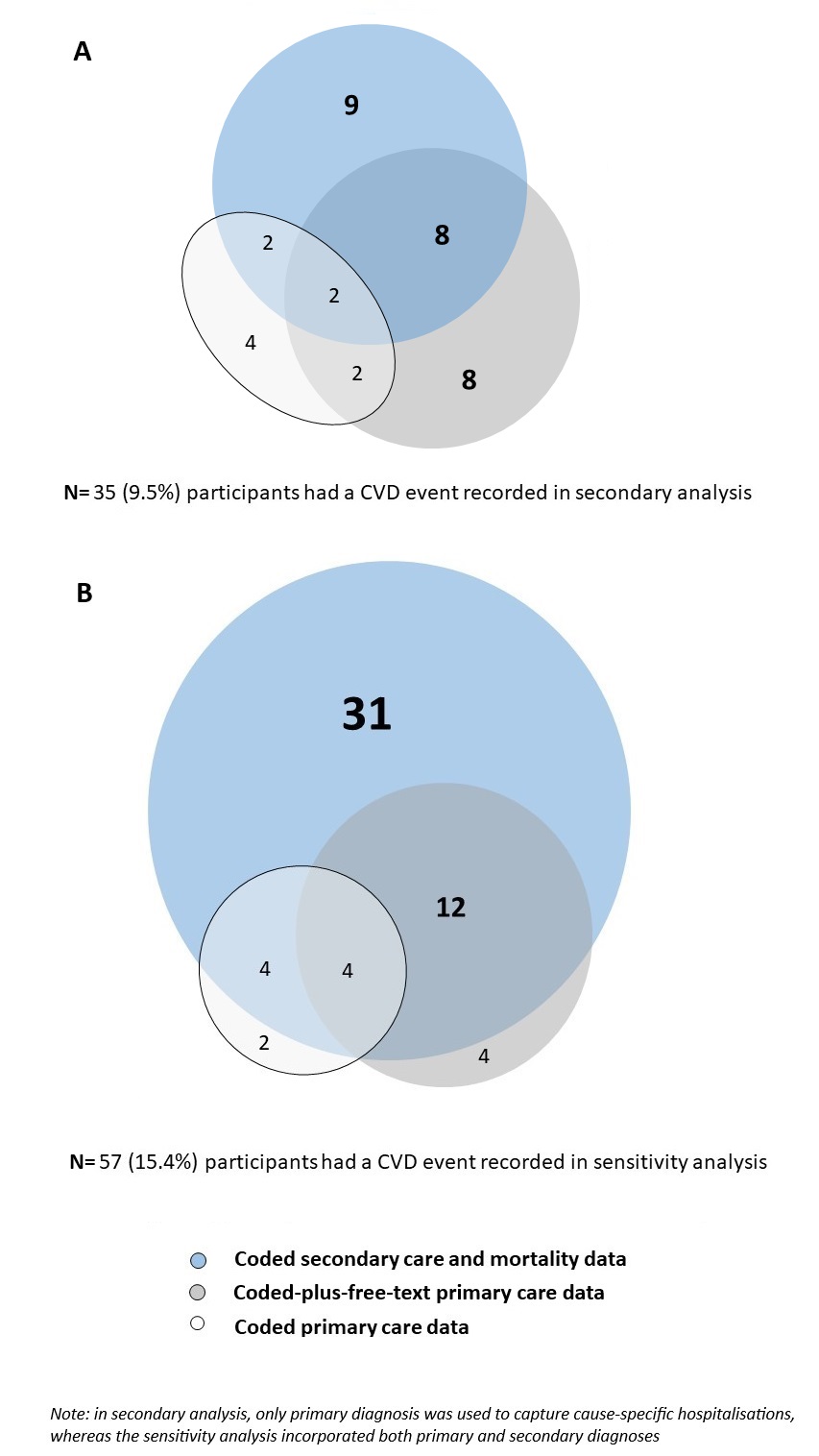
*

*Table S3. Time to event analyses of clinical outcomes at follow-up – sensitivity analysis, where all diagnosis codes that extracted from NHSE were used to define cause-specific hospitalisation*

|  | Medication reduction group | Usual care  Group | Adjusted hazard ratio^1^  (95% CI) | P-value |
| --- | --- | --- | --- | --- |
| Hospitalisation or death due to cardiovascular diseases | | | | |
| Coded primary care data | 5/185 (2.7%) | 5/184 (2.7%) | 0.89 (0.25 to 3.11) | 0.850 |
| Coded-plus-free-text primary care data | 9/185 (4.9%) | 11/184 (6%) | 0.79 (0.33 to 1.92) | 0.605 |
| Coded secondary care and mortality data^2^ | 26/185 (14.1%) | 25/184 (13.6%) | 0.96 (0.55 to 1.68) | 0.554 |
| Hospitalisation or death due to myocardial infarction | | | | |
| Coded primary care data | 1/185 (<1%) | 1/184 (<1%) | 1.06 (0.07 to 16.93) | 0.969 |
| Coded-plus-free-text primary care data | 4/185 (2.2%) | 3/184 (1.6%) | 1.27 (0.28 to 5.74) | 0.755 |
| Coded secondary care and mortality data | 8/185 (4.3%) | 9/184 (4.9%) | 0.83 (0.32 to 2.17) | 0.704 |
| Hospitalisation or death due to stroke | | | | |
| Coded primary care data | 1/185 (<1%) | 1/184 (<1%) | 0.66 (0.04 to 11.61) | 0.776 |
| Coded-plus-free-text primary care data | 3/185 (1.6%) | 7/184 (3.8%) | 0.41 (0.11 to 1.62) | 0.205 |
| Coded secondary care and mortality data | 4/185 (2.2%) | 7/184 (3.8%) | 0.50 (0.14 to 1.74) | 0.277 |
| Hospitalisation due to hypotension | | | | |
| Coded primary care data | 0 | 0 | - | - |
| Coded-plus-free-text primary care data | 0 | 0 | - | - |
| Coded secondary care and mortality data | 15/185 (8.1%) | 5/184 (2.7%) | **3.0 (1.09 to 8.28)** | **0.034** |
| Hospitalisation due to syncope |  |  |  |  |
| Coded primary care data | 1/185 (<1%) | 0/184 (0%) | - | - |
| Coded-plus-free-text primary care data | 2/185 (1.1%) | 0/184 (0%) | - | - |
| Coded secondary care and mortality data | 6/185 (3.2%) | 4/184 (2.2%) | 1.45 (0.40 to 5.16) | 0.571 |
| Hospitalisation due to falls | | | | |
| Coded primary care data | 3/185 (1.6%) | 3/184 (1.6%) | 1.06 (0.21 to 5.25) | 0.945 |
| Coded-plus-free-text primary care data | 12/185 (6.5%) | 13/184 (7.1%) | 0.90 (0.41 to 1.99) | 0.802 |
| Coded secondary care and mortality data | 19/185 (10.3%) | 14/184 (7.6%) | 1.31 (0.65 to 2.62) | 0.451 |
| Hospitalisation due to fracture | | | | |
| Coded primary care data | 5/185 (2.7%) | 1/184 (<1%) | 5.03 (0.59 to 43.29) | 0.141 |
| Coded-plus-free-text primary care data | 2/185 (1.1%) | 4/184 (2.2%) | 0.38 (0.07 to 2.18) | 0.280 |
| Coded secondary care and mortality data | 10/185 (5.4%) | 4/184 (2.2%) | 2.5 (0.78 to 8.03) | 0.122 |
| Hospitalisation due to electrolyte abnormalities | | | | |
| Coded primary care data | 2/185 (1.1%) | 0/184 (<1%) | - | - |
| Coded-plus-free-text primary care data | 1/185 (<1%) | 1/184 (<1%) | 1.06 (0.07 to 16.99) | 0.966 |
| Coded secondary care and mortality data | 21/185 (11.4%) | 16/184 (8.7%) | 1.31 (0.68 to 2.51) | 0.421 |
| Hospitalisation due to acute kidney injury | | | | |
| Coded primary care data | 0 | 0 | - | - |
| Coded-plus-free-text primary care data | 1/185 (<1%) | 1/184 (<1%) | 0.84 (0.05 to 14.15) | 0.904 |
| Coded secondary care and mortality data | 15/185 (8.1%) | 15/184 (8.2%) | 0.98 (0.48 to 2.02) | 0.964 |

*Note:*

*^1^Cox proportional hazards model adjusting baseline systolic blood pressure and intervention group as fixed effects. Hazard ration (HR) < 1 indicates favour to medication reduction group.*

*^2^ In this study, the Coded secondary care and mortality data represents the original OPTiMISE trial dataset*

Appendix I. List of ICD-10 codes to identify outcomes from NHS England data

| Condition | ICD- 10 code | Description |
| --- | --- | --- |
| Cardiovascular death | IX | Diseases of the circulatory system |
| Myocardial infarction | I21 | Acute myocardial infarction |
|  | I21.0 | Acute transmural myocardial infarction of anterior wall |
|  | I21.1 | Acute transmural myocardial infarction of inferior wall |
|  | I21.2 | Acute transmural myocardial infarction of other sites |
|  | I21.3 | Acute transmural myocardial infarction of unspecified site |
|  | I21.4 | Acute subendocardial myocardial infarction |
|  | I21.9 | Acute myocardial infarction, unspecified |
|  | I22 | Subsequent myocardial infarction |
|  | I22.0 | Subsequent myocardial infarction of anterior wall |
|  | I22.1 | Subsequent myocardial infarction of inferior wall |
|  | I22.8 | Subsequent myocardial infarction of other sites |
|  | I22.9 | Subsequent myocardial infarction of unspecified site |
|  | I24 | Other acute ischaemic heart diseases |
|  | I24.8 | Other forms of acute ischaemic heart disease |
|  | I24.9 | Acute ischaemic heart disease, unspecified |
| Heart Failure | I50 | Heart failure |
|  | I50.0 | Congestive heart failure |
|  | I50.1 | Left ventricular failure |
|  | I50.9 | Heart failure, unspecified |
| Stroke | I60 | Subarachnoid haemorrhage |
|  | I60.0 | Subarachnoid haemorrhage from carotid siphon and bifurcation |
|  | I60.1 | Subarachnoid haemorrhage from middle cerebral artery |
|  | I60.2 | Subarachnoid haemorrhage from anterior communicating artery |
|  | I60.3 | Subarachnoid haemorrhage from posterior communicating artery |
|  | I60.4 | Subarachnoid haemorrhage from basilar artery |
|  | I60.5 | Subarachnoid haemorrhage from vertebral artery |
|  | I60.6 | Subarachnoid haemorrhage from other intracranial arteries |
|  | I60.7 | Subarachnoid haemorrhage from intracranial artery, unspecified |
|  | I60.8 | Other subarachnoid haemorrhage |
|  | I60.9 | Subarachnoid haemorrhage, unspecified |
|  | I61 | Intracerebral haemorrhage |
|  | I61.0 | Intracerebral haemorrhage in hemisphere, subcortical |
|  | I61.1 | Intracerebral haemorrhage in hemisphere, cortical |
|  | I61.2 | Intracerebral haemorrhage in hemisphere, unspecified |
|  | I61.3 | Intracerebral haemorrhage in brain stem |
|  | I61.4 | Intracerebral haemorrhage in cerebellum |
|  | I61.5 | Intracerebral haemorrhage, intraventricular |
|  | I61.6 | Intracerebral haemorrhage, multiple localized |
|  | I61.8 | Other intracerebral haemorrhage |
|  | I61.9 | Intracerebral haemorrhage, unspecified |
|  | I62 | Other nontraumatic intracranial haemorrhage |
|  | I62.9 | Intracranial haemorrhage (nontraumatic), unspecified |
|  | I63 | Cerebral infarction |
|  | I63.0 | Cerebral infarction due to thrombosis of precerebral arteries |
|  | I63.1 | Cerebral infarction due to embolism of precerebral arteries |
|  | I63.2 | Cerebral infarction due to unspecified occlusion or stenosis of precerebral arteries |
|  | I63.3 | Cerebral infarction due to thrombosis of cerebral arteries |
|  | I63.4 | Cerebral infarction due to embolism of cerebral arteries |
|  | I63.5 | Cerebral infarction due to unspecified occlusion or stenosis of cerebral arteries |
|  | I63.6 | Cerebral infarction due to cerebral venous thrombosis, nonpyogenic |
|  | I63.8 | Other cerebral infarction |
|  | I63.9 | Cerebral infarction, unspecified |
|  | I64 | Stroke, not specified as haemorrhage or infarction |
| Hypotension | I95 | Hypotension |
|  | I95.0 | Idiopathic hypotension |
|  | I95.1 | Orthostatic hypotension |
|  | I95.2 | Hypotension due to drugs |
|  | I95.8 | Other hypotension |
|  | I95.9 | Hypotension, unspecified |
| Syncope | R55 | Syncope and collapse |
| Fracture | S02 | Fracture of skull and facial bones |
|  | S02.0 | Fracture of vault of skull |
|  | S02.1 | Fracture of base of skull |
|  | S02.2 | Fracture of nasal bones |
|  | S02.3 | Fracture of orbital floor |
|  | S02.4 | Fracture of malar and maxillary bones |
|  | S02.5 | Fracture of tooth |
|  | S02.6 | Fracture of mandible |
|  | S02.7 | Multiple fractures involving skull and facial bones |
|  | S02.8 | Fractures of other skull and facial bones |
|  | S02.9 | Fracture of skull and facial bones, part unspecified |
|  | S12 | Fracture of neck |
|  | S12.0 | Fracture of first cervical vertebra |
|  | S12.1 | Fracture of second cervical vertebra |
|  | S12.2 | Fracture of other specified cervical vertebra |
|  | S12.7 | Multiple fractures of cervical spine |
|  | S12.8 | Fracture of other parts of neck |
|  | S12.9 | Fracture of neck, part unspecified |
|  | S22 | Fracture of rib(s), sternum and thoracic spine |
|  | S22.0 | Fracture of thoracic vertebra |
|  | S22.1 | Multiple fractures of thoracic spine |
|  | S22.2 | Fracture of sternum |
|  | S22.3 | Fracture of rib |
|  | S22.4 | Multiple fractures of ribs |
|  | S22.5 | Flail chest |
|  | S22.8 | Fracture of other parts of bony thorax |
|  | S22.9 | Fracture of bony thorax, part unspecified |
|  | S32 | Fracture of lumbar spine and pelvis |
|  | S32.0 | Fracture of lumbar vertebra |
|  | S32.1 | Fracture of sacrum |
|  | S32.2 | Fracture of coccyx |
|  | S32.3 | Fracture of ilium |
|  | S32.4 | Fracture of acetabulum |
|  | S32.5 | Fracture of pubis |
|  | S32.7 | Multiple fractures of lumbar spine and pelvis |
|  | S32.8 | Fracture of other and unspecified parts of lumbar spine and pelvis |
|  | S42 | Fracture of shoulder and upper arm |
|  | S42.0 | Fracture of clavicle |
|  | S42.1 | Fracture of scapula |
|  | S42.2 | Fracture of upper end of humerus |
|  | S42.3 | Fracture of shaft of humerus |
|  | S42.4 | Fracture of lower end of humerus |
|  | S42.7 | Multiple fractures of clavicle, scapula and humerus |
|  | S42.8 | Fracture of other parts of shoulder and upper arm |
|  | S42.9 | Fracture of shoulder girdle, part unspecified |
|  | S52 | Fracture of forearm |
|  | S52.0 | Fracture of upper end of ulna |
|  | S52.1 | Fracture of upper end of radius |
|  | S52.2 | Fracture of shaft of ulna |
|  | S52.3 | Fracture of shaft of radius |
|  | S52.4 | Fracture of shafts of both ulna and radius |
|  | S52.5 | Fracture of lower end of radius |
|  | S52.6 | Fracture of lower end of both ulna and radius |
|  | S52.7 | Multiple fractures of forearm |
|  | S52.8 | Fracture of other parts of forearm |
|  | S52.9 | Fracture of forearm, part unspecified |
|  | S62 | Fracture at wrist and hand level |
|  | S62.0 | Fracture of navicular [scaphoid] bone of hand |
|  | S62.1 | Fracture of other carpal bone(s) |
|  | S62.2 | Fracture of first metacarpal bone |
|  | S62.3 | Fracture of other metacarpal bone |
|  | S62.4 | Multiple fractures of metacarpal bones |
|  | S62.5 | Fracture of thumb |
|  | S62.6 | Fracture of other finger |
|  | S62.7 | Multiple fractures of fingers |
|  | S62.8 | Fracture of other and unspecified parts of wrist and hand |
|  | S72 | Fracture of femur |
|  | S72.0 | Fracture of neck of femur |
|  | S72.1 | Pertrochanteric fracture |
|  | S72.2 | Subtrochanteric fracture |
|  | S72.3 | Fracture of shaft of femur |
|  | S72.4 | Fracture of lower end of femur |
|  | S72.7 | Multiple fractures of femur |
|  | S72.8 | Fractures of other parts of femur |
|  | S72.9 | Fracture of femur, part unspecified |
|  | S82 | Fracture of lower leg, including ankle |
|  | S82.0 | Fracture of patella |
|  | S82.1 | Fracture of upper end of tibia |
|  | S82.2 | Fracture of shaft of tibia |
|  | S82.3 | Fracture of lower end of tibia |
|  | S82.4 | Fracture of fibula alone |
|  | S82.5 | Fracture of medial malleolus |
|  | S82.6 | Fracture of lateral malleolus |
|  | S82.7 | Multiple fractures of lower leg |
|  | S82.8 | Fractures of other parts of lower leg |
|  | S82.9 | Fracture of lower leg, part unspecified |
|  | S92 | Fracture of foot, except ankle |
|  | S92.0 | Fracture of calcaneus |
|  | S92.1 | Fracture of talus |
|  | S92.2 | Fracture of other tarsal bone(s) |
|  | S92.3 | Fracture of metatarsal bone |
|  | S92.4 | Fracture of great toe |
|  | S92.5 | Fracture of other toe |
|  | S92.7 | Multiple fractures of foot |
|  | S92.9 | Fracture of foot, unspecified |
|  | T02 | Fractures involving multiple body regions |
|  | T02.0 | Fractures involving head with neck |
|  | T02.1 | Fractures involving thorax with lower back and pelvis |
|  | T02.2 | Fractures involving multiple regions of one upper limb |
|  | T02.3 | Fractures involving multiple regions of one lower limb |
|  | T02.4 | Fractures involving multiple regions of both upper limbs |
|  | T02.5 | Fractures involving multiple regions of both lower limbs |
|  | T02.6 | Fractures involving multiple regions of upper limb(s) with lower limb(s) |
|  | T02.7 | Fractures involving thorax with lower back and pelvis with limb(s) |
|  | T02.8 | Fractures involving other combinations of body regions |
|  | T02.9 | Multiple fractures, unspecified |
|  | T08 | Fracture of spine, level unspecified |
|  | T10 | Fracture of upper limb, level unspecified |
|  | T12 | Fracture of lower limb, level unspecified |
|  | T14.2 | Fracture of unspecified body region |
| Fall | W01 | Fall on same level from slipping, tripping and stumbling |
|  | W05 | Fall involving wheelchair |
|  | W06 | Fall involving bed |
|  | W07 | Fall involving chair |
|  | W08 | Fall involving other furniture |
|  | W10 | Fall on and from stairs and steps |
|  | W17 | Other fall from one level to another |
|  | W18 | Other fall on same level |
|  | W19 | Unspecified fall |
| Dementia | F00 | Dementia in Alzheimer disease |
|  | F00.0 | Dementia in Alzheimer disease with early onset |
|  | F00.1 | Dementia in Alzheimer disease with late onset |
|  | F00.2 | Dementia in Alzheimer disease, atypical or mixed type |
|  | F00.9 | Dementia in Alzheimer disease, unspecified |
|  | F01 | Vascular dementia |
|  | F01.0 | Vascular dementia of acute onset |
|  | F01.1 | Multi-infarct dementia |
|  | F01.2 | Subcortical vascular dementia |
|  | F01.3 | Mixed cortical and subcortical vascular dementia |
|  | F01.8 | Other vascular dementia |
|  | F01.9 | Vascular dementia, unspecified |
|  | F02 | Dementia in other diseases classified elsewhere |
|  | F02.0 | Dementia in Pick disease |
|  | F02.1 | Dementia in Creutzfeldt-Jakob disease |
|  | F02.2 | Dementia in Huntington disease |
|  | F02.3 | Dementia in Parkinson disease |
|  | F02.4 | Dementia in human immunodeficiency virus [HIV] disease |
|  | F02.8 | Dementia in other specified diseases classified elsewhere |
|  | F03 | Unspecified dementia |
| Acute kidney injury | S37.0 | Injury of kidney |
|  | N19 | Unspecified kidney failure |
|  | N17 | Acute renal failure |
|  | N17.0 | Acute renal failure with tubular necrosis |
|  | N17.1 | Acute renal failure with acute cortical necrosis |
|  | N17.2 | Acute renal failure with medullary necrosis |
|  | N17.8 | Other acute renal failure |
|  | N17.9 | Acute renal failure, unspecified |
|  | 866 | kidney injury* |
|  | 866 | kidney injury-closed* |
|  | 866 | kidney injury nos-closed |
|  | 586 | renal failure nos |
|  | 584 | acute renal failure* |
|  | 584.5 | ac kidny fail, tubr necr |
|  | 584.6 | ac kidny fail, cort necr |
|  | 584.7 | ac kidny fail, medu necr |
|  | 584.8 | acute kidney failure nec |
|  | 584.9 | acute kidney failure nos |
| Electrolyte abnormalities | 276 | fluid/electrolyte dis* |
|  | 276 | hyperosmolality |
|  | 276.1 | hyposmolality |
|  | 276.2 | acidosis |
|  | 276.3 | alkalosis |
|  | 276.4 | mixed acid-base bal dis |
|  | 276.5 | hypovolemia# |
|  | 276.6 | fluid overload# |
|  | 276.7 | hyperpotassemia |
|  | 276.8 | hypopotassemia |
|  | 276.9 | electrolyt/fluid dis nec |
|  | E87 | Other disorders of fluid, electrolyte and acid-base balance |
|  | E87.0 | Hyperosmolality and hypernatraemia |
|  | E87.1 | Hypo-osmolality and hyponatraemia |
|  | E87.2 | Acidosis |
|  | E87.3 | Alkalosis |
|  | E87.4 | Mixed disorder of acid-base balance |
|  | E87.5 | Hyperkalaemia |
|  | E87.6 | Hypokalaemia |
|  | E87.7 | Fluid overload |
|  | E87.8 | Other disorders of electrolyte and fluid balance, not elsewhere classified |
